# Adenosine Kinase of *T. b. rhodesiense* Identified as the Putative Target of 4-[5-(4-phenoxyphenyl)-2*H*-pyrazol-3-yl]morpholine Using Chemical Proteomics

**DOI:** 10.1371/journal.pntd.0000506

**Published:** 2009-08-25

**Authors:** Sabine Kuettel, Marc Mosimann, Pascal Mäser, Marcel Kaiser, Reto Brun, Leonardo Scapozza, Remo Perozzo

**Affiliations:** 1 Pharmaceutical Biochemistry Group, School of Pharmaceutical Sciences, University of Geneva, University of Lausanne, Geneva, Switzerland; 2 Institute of Cell Biology, University of Bern, Bern, Switzerland; 3 Parasite Chemotherapy, Swiss Tropical Institute, Basel, Switzerland; University of Bern, Switzerland

## Abstract

**Background:**

Human African trypanosomiasis (HAT), a major parasitic disease spread in Africa, urgently needs novel targets and new efficacious chemotherapeutic agents. Recently, we discovered that 4-[5-(4-phenoxyphenyl)-2*H*-pyrazol-3-yl]morpholine (compound **1**) exhibits specific antitrypanosomal activity with an IC_50_ of 1.0 µM on *Trypanosoma brucei rhodesiense* (*T. b. rhodesiense*), the causative agent of the acute form of HAT.

**Methodology/Principal Findings:**

In this work we show adenosine kinase of *T. b. rhodesiense* (TbrAK), a key enzyme of the parasite purine salvage pathway which is vital for parasite survival, to be the putative intracellular target of compound **1** using a chemical proteomics approach. This finding was confirmed by RNA interference experiments showing that down-regulation of adenosine kinase counteracts compound **1** activity. Further chemical validation demonstrated that compound **1** interacts specifically and tightly with TbrAK with nanomolar affinity, and *in vitro* activity measurements showed that compound **1** is an enhancer of TbrAK activity. The subsequent kinetic analysis provided strong evidence that the observed hyperactivation of TbrAK is due to the abolishment of the intrinsic substrate-inhibition.

**Conclusions/Significance:**

The results suggest that TbrAK is the putative target of this compound, and that hyperactivation of TbrAK may represent a novel therapeutic strategy for the development of trypanocides.

## Author Summary

Human African trypanosomiasis (HAT), a devastating and fatal parasitic disease endemic in sub-Saharan Africa, urgently needs novel targets and efficacious chemotherapeutic agents. Recently, we discovered that 4-[5-(4-phenoxyphenyl)-2*H*-pyrazol-3-yl]morpholine exhibits specific antitrypanosomal activity toward *T. b. rhodesiense*, the causative agent of the acute form of HAT. Here we applied a chemical proteomics approach to find the cellular target of this compound. Adenosine kinase, a key enzyme of the parasite purine salvage pathway, was isolated and identified as compound binding partner. Direct binding assays using recombinant protein, and tests on an adenosine kinase knock-down mutant of the parasite produced by RNA interference confirmed TbrAK as the putative target. Kinetic analyses showed that the title compound is an activator of adenosine kinase and that the observed hyperactivation of TbrAK is due to the abolishment of the intrinsic substrate-inhibition. Whereas hyperactivation as a mechanism of action is well known from drugs targeting cell signaling, this is a novel and hitherto unexplored concept for compounds targeting metabolic enzymes, suggesting that hyperactivation of TbrAK may represent a novel therapeutic strategy for the development of trypanocides.

## Introduction

Trypanosomiases belong to the major parasitic diseases spread throughout the world. Human African Trypanosomiasis (HAT; sleeping sickness) is a vector-borne parasitic disease transmitted by a protozoan parasite of the genus *Trypanosoma* via the bites of infected tsetse flies. Two different forms of HAT are known. The chronic form is caused by *Trypanosoma brucei gambiense (T. b. gambiense)* infection whereas *Trypanosoma brucei rhodesiense (T. b. rhodesiense)* is responsible for the acute form of the disease. Both forms of HAT develop in two stages. After the bite, parasites first distribute to the blood, lymph and peripheral organs (stage 1), then spread to the central nervous system (stage 2) where they cause serious neurological disorders. Leaving infected people untreated, HAT is invariably fatal. Today, sleeping sickness threatens millions of people in 36 countries of sub-Saharan Africa [1],[2]. The estimated number of deaths annually is currently between 50’000 and 70’000 [3].

Four drugs (pentamidine, melarsoprol, eflornithine and suramin) are registered for the treatment of sleeping sickness and provided free of charge to endemic countries through a World Health Organization (WHO) private partnership. The type of treatment depends on the stage of the disease. Pentamidine and suramin are used for first stage treatment of *T. b. gambiense* and *T. b. rhodesiense* sleeping sickness, respectively. The drugs capable to treat the second stage infections are more toxic and complicated to administer, and they need to cross the blood-brain barrier to reach the parasites within the central nervous system. Melarsoprol, which is effective against both forms of HAT, derives from arsenic and has many severe side effects, the most dramatic (prevalence 5 to 15%) being a reactive encephalopathy (encephalopathic syndrome) which can be fatal in 3–10% of affected patients. An increase of resistance to the drug has been observed in several foci particularly in central Africa. Eflornithine is less toxic than melarsoprol, but it is only effective against *T. b. gambiense* sleeping sickness, and a strict and complicated regimen has to be applied. It is evident that therapy of HAT relies on few drugs which are associated with severe side effects. There has been a revival of drug research and development regarding HAT compared to the last 15 years, and a number of drug development projects are currently ongoing. Unfortunately, the development of the only compound (pafuramidine) having advanced to phase III clinical trials [4],[5] for stage one treatment was discontinued [6]. Thus, novel targets and new efficacious chemotherapeutic agents are urgently needed.

**Figure 1:**
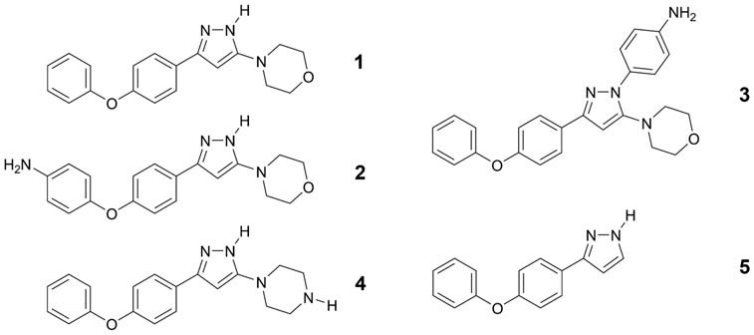
Chemical structures of compounds used in this study. The title compound 4-[5-(4-phenoxyphenyl)-2H-pyrazol-3-yl]morpholine (1) and its amino substituted derivatives 4-[4-(5-morpholine-4-yl-1H-pyrazol-3-yl)-phenoxy]phenylamine (2), 4-[5-morpholine-4-yl-3-(4-phenoxyphenyl)-pyrazol-1-yl]phenylamine (3) and 1-[5-(4-phenoxyphenyl-2H-pyrazol-3-yl]piperazine (4) are presented. 3-(4-phenoxyphenyl)-1H-pyrazole (5) showed very low antiparasitic activity (IC_50_ of >65 µM) as well as general toxicity (IC_50_ of >136 µM), therefore it was used as negative control for the biochemical, biophysical and compound sensitivity tests.

Recently we reported the synthesis and evaluation of new 4-[5-(4-phenoxy-phenyl-2*H*-pyrazol-3-yl]morpholine derivatives against several parasites [7]. One of the compounds, 4-[5-(4-phenoxy-phenyl-2*H*-pyrazol-3-yl]morpholine (Fig. 1, compound **1**), exhibited good activity toward *T. b. rhodesiense* with an IC_50_ of 1 µM and low cytotoxicity [7]. This finding prompted us to address the question regarding the cellular target and the molecular mechanism underlying the observed toxicity toward the parasite. To this end, here we report a chemical proteomics approach that led to the identification of adenosine kinase as the putative target. Subsequent biochemical and biophysical characterization with respect to compound **1** binding as well as drug sensitivity tests on the corresponding knock-down strain allowed its validation and suggested hyperactivation of adenosine kinase as the molecular mechanism underlying the biological activity.

## Methods

### Preparation of *T. b. rhodesiense* lysate

The parasites collected from mouse blood were lysed with lysis buffer consisting of 20 mM Hepes, 150 mM NaCl, 1% Triton X-100, 2 mM tris(2-carboxyethyl)phosphine (TCEP), 10% glycerol, 1 µl/ml protease inhibitor cocktail, at pH 7.5. To this end, the suspension of the purified cells (2.56×10^9^ trypanosomes) in 160 µl PBS were treated with 40 µl lysis buffer concentrate (five fold). After short intervals of sonication the mixture was centrifuged for 10 min at 14’000 g, the supernatant was recovered and stored in aliquots at -20°C. The total protein concentration was measured using Bradford dye assay [8], and the lysate was further diluted to the desired concentration using lysis buffer. All protocols and procedures used in the current study were reviewed and approved by the local veterinary authorities of the Canton Basel-Stadt (Switzerland).

### Affinity chromatography and identification by mass spectrometry

Compounds **2**, **3**, and **4** (Fig. 1) were immobilized via the primary or secondary amino groups (Fig. S1) on epoxy-activated agarose providing a 12-atoms spacer (1,4-bis(2∶3-epoxypropoxy)butane). Swollen and thoroughly washed matrix was resuspended in two to three volumes of 12.5–50 mM ligand dissolved in 50% DMF/50 mM Na_2_CO_3_, 50 mM NaCl, pH 9.5. Coupling was performed for 16–72 h at 25–40°C. After 4 to 5 washes with 50% DMF/50 mM Na_2_CO_3_, 50 mM NaCl, pH 9.5, remaining reactive groups were blocked with 1 M ethanolamine (pH 8) and thoroughly washed with low/high pH buffers. A control matrix was prepared without ligand and treated as described above. Direct absorbance scans of the immobilized ligand on the matrix resuspended in 50% glycerol solution (v/v) clearly confirmed successful coupling (data not shown). The amount of inhibitor bound to the matrix was determined by back calculation of amount of compound applied and amount recovered by UV determination. Routinely 20–45 µmol/ml compound were bound. The resin was incubated in 2.5 volumes of *T. b. rhodesiense* total cell lysate (2 mg/ml protein) and incubated at 4°C for 2 hours. After washing with lysis buffer (four times), the matrix was heated at 95°C for 5 min with Laemmli sample buffer, directly loaded and separated by 12% SDS-PAGE. Resolved proteins were visualized by silver staining. The control matrix was incubated with the same amount of lysate and treated equally. Proteins bands were excised from the gels and fragments resulting from trypsin digestion were analyzed by LC/ESI/MS/MS-QTOF mass spectrometry. Database searches were performed by using the ProteinLynx Global Server (all species) and Mascot (other eukaryotes) search programs. A score >46 indicates identity (p<0.05).

### HPLC activity assay for TbrAK

TbrAK catalyzes the ATP-dependent phosphorylation of the 5′-hydroxyl moiety of adenosine to form AMP and ADP. The activity assay was based on HPLC separation of substrate and products (adenosine, AMP, ADP, and ATP). The mobile phase consisted of 35 mM KH_2_PO_4_, 6 mM tetrabutylammonium hydrogensulfate, (pH 7.0, adjusted with KOH), 125 mM EDTA, and 1% (v/v) acetonitrile. The separation was performed isocratically at a flow-rate of 1.5 ml/min and monitored at 254 nm. The standard reaction in 75 µl total volume containing 0.5 mM ATP, 0.5 mM adenosine, 0.5 mM MgCl_2_, 1% DMSO in buffer E1 (20 mM Hepes, 150 mM NaCl, pH 7.0) was started with the addition of 2 µg TbrAK (final concentration 0.7 µM) and incubated at 37°C for 10 min while shaking vigorously (600 rpm). To measure the influence of compound **1**–**5** on TbrAK activity, DMSO was replaced in the assay mixture by compound **1**–**5** so that the final concentration of 1% DMSO was maintained while the concentration of compound could be varied in the range of 0–1 mM depending on solubility. All reactions were stopped by adding 75 µl mobile phase. 50 µl of the sample volume were injected for analysis and the resulting ADP/ATP ratios were calculated and used as a measure of activity. For comparative reasons the activity recorded in absence of compound was set to 100%. The mean of three independent measurements are reported.

### Calorimetric measurements

Isothermal titration calorimetry (ITC) was employed to elucidate the binding affinity of compound **1** toward TbrAK. All solutions were degassed for 5 min with gentle stirring under vacuum. TbrAK was dialyzed into buffer E2 (20 mM Tris, 150 mM NaCl, pH 7.5, 5% glycerol), diluted to 7 µM and supplemented with 1% DMSO, filtered and filled into the sample cell. The ligand solution was prepared by diluting a stock solution of compound **1** or **5** in DMSO with buffer E2 to give a final concentration of 150 µM and 1% DMSO. The final ligand solution was filtered before use. A titration experiment consisted of a first control injection of 1 µl followed by 27 injections, each of 10 µl and 20 s duration, with a 4-min interval in between. Raw data were collected, corrected for ligand heats of dilution, integrated and fit to the two-set non-interacting binding sites model using the Origin® software supplied with the instrument. The measurements were performed at least in duplicate.

### Radiometric assay for kinetic study of TbrAK

Kinetic constants for adenosine phosphorylation by TbrAK were obtained by monitoring the conversion of [2-^3^H]adenosine to [2-^3^H]AMP. Reactions were executed at 37°C in a final volume of 30 µl containing 20 mM Hepes (pH 7.0), 50 mM NaCl, 167 µM ATP, 167 µM MgCl_2_, 0.34% BSA, and 0.34% DMSO. To measure the influence of compound **1** to kinetic parameters, DMSO was omitted in the assay mixture and **1** was added from a 10 mM stock solution in DMSO to give a final concentration of 33.4 µM and 0.34% DMSO. The concentration of [2-^3^H]adenosine (0.1–12 µM) was chosen in consideration of Michaelis–Menten conditions for initial velocity measurements. The reaction was started by the addition of 1 ng (0.88 nM) TbrAK. The reactions were incubated and 5 µl aliquots were spotted on 5 mm diameter DE81-cellulose disks placed in a 96-well plate to stop the reaction. The disks were washed 3 times with 250 µl 5 mM ammonium formate, once with H_2_O, transferred to scintillation vials, and then soaked with 2 ml of a displacement solution (100 mM HCl, 200 mM KCl) and gently shaken for 1 minute to elute the phosphorylated products [9]. After adding 10 ml scintillation liquid, the samples were counted in scintillation counter. K_m_ and V_max_ with respect to adenosine and K_i_ for adenosine substrate-inhibition in absence and presence of compound **1** were determined by non-linear fit of the data to the substrate-inhibition model described elsewhere [10]. The results are based on three independent series with each data point measured in triplicates.

### Genetic knock-down of TbAK and drug sensitivity tests

The construction of the *T. b. brucei* adenosine kinase gene knock-down mutant has been described recently [11]. Expression of a stem-loop construct targeting TbAK was induced by addition of 10 µg/ml tetracycline to the medium. Down-regulation of TbAK expression was verified by Northern blotting. Parasites were cultivated and drug sensitivity was tested using the Alamar blue test [12]. IC_50_ values were calculated by nonlinear fitting to the sigmoidal dose-response curve using Origin® software. The assays were performed in triplicates for at least four times.

### Other methods

Origin of supplies as well as protocols for purification of recombinant proteins, the spectroscopic assay for TbrGAPDH, the radiometric assay for TbrAK, and the thermal denaturation assay are described in the Supporting Information (Text S1).

## Results

### Compound choice and immobilization

In order to isolate and identify the intracellular target(s) of compound **1** in *T. b. rhodesiense* we decided to immobilize the compound and to isolate their target(s) by an affinity chromatography approach. The binding mode of **1** to the putative target(s) being unknown, we immobilized three derivatives of compound **1** (Fig. 1, compound **2**–**4**), each containing an additional primary or secondary amino group at different positions [7], and linked them to epoxy-activated agarose. The primary aromatic amine in compound **2** and **3**, and the secondary amino group in the piperazine moiety of compound **4** react with the terminal epoxy group of the activated agarose, providing an uncharged, hydrophilic and very stable 12-atoms spacer (see Fig. S1). The direct linkage of compound **1** to epoxy-activated agarose via the pyrazole moiety was not likely to occur under the applied experimental conditions due to the weak nucleophilicity of the heterocycle for epoxide-based alkylation which in general requires rather drastic conditions, i.e. prolonged heating at high temperature with the concomitant use of a strong base [13]. Direct absorbance scans of the immobilized ligand on the matrix resuspended in 50% glycerol solution (v/v) confirmed successful coupling (data not shown). Routinely 20–45 µmol/ml compound were bound. Our recent evaluation of compound **1** and its derivatives clearly indicated the morpholine moiety to be important for the biological effect [7] which is corroborated by the fact that compound **5** (morpholine removed) only exhibits very low antiparasitic activity (IC_50_ of >65 µM). Therefore compound **5** was chosen as negative control.

### Immobilized compounds bind TbrGAPDH and TbrAK

**Figure 2:**
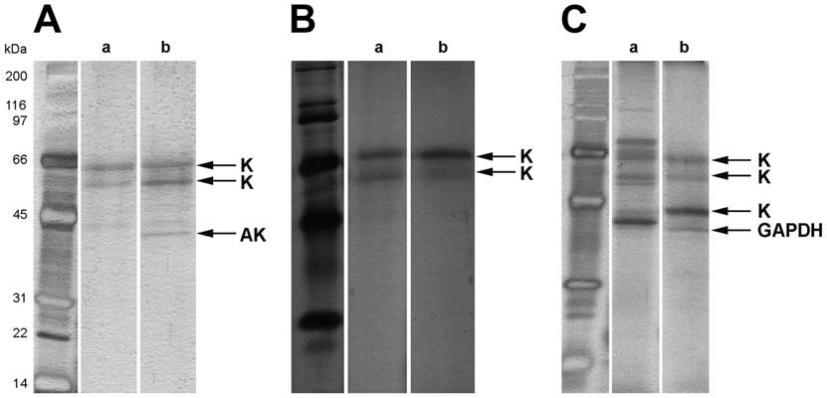
SDS-PAGE (12%) analysis of proteins retained by the affinity matrices. Panel A: Matrix linked with derivative 2. Panel B: Matrix linked with derivative 3. Panel C: Matrix linked with derivative 4. Samples of *T. b. rhodesiense* lysate (200 µg total protein content) were incubated with either 40 µl control beads (lanes a) or 40 µl affinity beads (lanes b). Proteins retained by the matrices were directly separated by SDS-PAGE. Arrows indicate protein bands resulting from the compound derived matrices. AK: adenosine kinase of *T. b. rhodesiense*; GAPDH: glycosomal glyceraldehyde-3-phosphate dehydrogenase of *T. b. rhodesiense*; K: human keratin. Molecular mass markers are shown on the left.

The total parasite lysate was prepared, loaded on the matrices and unbound material was removed as outlined in the Methods section. The bound proteins were separated by SDS-PAGE and visualized by silver-staining (Fig. 2). The use of compound **2** derived matrix led to the detection of three distinct protein bands, two of which at higher molecular weights also being present in the control (Fig. 2A). Trypsin digestion and peptide sequencing by LC/ESI/MS/MS-QTOF mass spectrometry identified the specific band at ∼40 kDa as adenosine kinase of *T. b. rhodesiense* (TbrAK, expected size of 38.1 kDa; score 141), whereas the other two corresponded to human keratin (score 392 and 752). Affinity matrix prepared with compound **3** did not isolate a protein different to the back ground signals identified as human keratin (Fig. 2B). In contrast, immobilized derivative **4** behaved differently, and more distinct proteins were isolated. Subsequent protein analysis found human keratin in most cases. One distinct protein band not visible in the control was identified as glycosomal *T. b. rhodesiense* glyceraldehyde-3-phosphate dehydrogenase (TbrGAPDH, expected size of 39.3 kDa; score 87) (Fig. 2C). Protein bands isolated on the control matrices were considered to bind unspecifically and were not further examined.

### Production of recombinant TbrGAPDH and TbrAK

For chemical validation of both TbrGAPDH and TbrAK, proteins were produced recombinantly in sufficient amounts. The *gapdh* gene, kindly provided by Prof. Paul A. M. Michels (University of Louvain, Brussels, Belgium), was subcloned into an expression vector that yielded 10–15 mg of highly pure (>99%) TbrGAPDH per liter culture (Fig. S2A). Since the *ak* is a tandem gene coding for two almost identical (99% identity, 4 out of 345 amino acids different) adenosine kinases (SwissProt entries Q584S0 and Q584S6) it was necessary to determine which one had been isolated. The detailed analysis of the fragments resulting from the trypsin digestion revealed that peptide DIESTVLATK can be unambiguously allocated to the Q584S0 sequence, thus this isoform of TbrAK was cloned, expressed and purified to homogeneity and resulted in 40–50 mg of soluble and highly pure (>99%) TbrAK per liter culture (Fig. S2B). Both TbrGAPDH and TbrAK were active and could be used for subsequent analyses.

### Compound 1 enhances TbrAK activity

**Figure 3:**
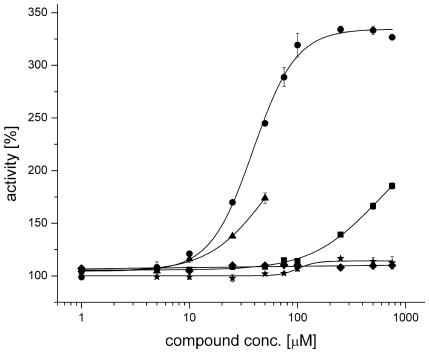
Concentration dependence of the activation effect for compounds 1 to 5 in the range of 1–750 µM. Increasing concentrations of compound 1 (•) gives a sigmoid saturation curve for TbrAK activation, yielding an EC_50_ value of 38.9±0.9 µM. A similar trend is observed for compound 2 (▴), but due to limited solubility at concentrations >50 µM the EC_50_ could not be determined. Compound 4 (▪) started activating TbrAK in a concentration dependent manner without reaching a maximum at the solubility limiting concentration of 750 µM. Compound 3 (♦) and the control compound 5 (★) did not activate TbrAK under identical conditions. Values are reported as % activity derived from the transformation rate. For comparative reasons the activity recorded in absence of compound was set to 100%. Standard deviations are represented by vertical bars. The mean of three independent measurements is reported.

TbrAK activity in absence and presence of the compounds was analyzed using an HPLC protocol that allowed the separation of adenosine, AMP, ADP, and ATP. Surprisingly, the assay revealed a 2.5-fold (absolute value: 245±3%) and 1.7-fold (174±5%) increase of enzyme activity in presence of 50 µM of compound **1** and **2**, respectively (Fig. 3; Fig. S3). A less pronounced but still significant effect (two-tailed t-test, p<0.01) was observed for compound **3** and **4** at 50 µM with an apparent increase of activity to 110±1% and 109±1%, respectively. The negative control (compound **5**) did not have this activating effect on TbrAK activity (see Fig. S3). To validate the activity measured at a single concentration, the concentration dependence of the effect was analyzed. The hyperactivation effect is found to be concentration dependent for compounds **1**, **2** and **4** (Fig. 3). The results clearly show that under the experimental conditions a half maximal effective concentration (EC_50_) of 38.9±0.9 µM for compound **1** is determined. Although the solubility limits prevented any EC_50_ determination regarding compounds **2** and **4** (Fig. 3), the concentration dependence, thus the specificity of the effect is proven also for these compounds. Interestingly, although compound **3** appeared to activate TbrAK at a low level (110±1%) it does not exhibit any concentration dependence when assayed up to concentrations corresponding to its maximum solubility, thus behaved like the inactive control compound **5** (Fig. 3).

The EC_50_ of found for compound **1** seemed to contrast the IC_50_ of 1.0 µM on the parasite. We reasoned that the experimental conditions of the HPLC assay may be at the origin of the high EC_50_ value, thus we analyzed the activating effect of compound **1** using the more sensitive radiometric assay, which allowed the analysis at low substrate and enzyme concentrations (Supporting Information Text S1), as an orthogonal method to the HPLC assay. Indeed, the hyperactivation effect was concentration dependent with an EC_50_ of 38±12 nM for compound **1** (Fig. S4). This value is well in line with the observed affinity (K_D_ of 75±20 nM and 497±34 nM for the high and low affinity binding site, respectively) of compound **1** towards TbrAK, and the observed IC_50_ of 1.0 µM in the *in-vitro* whole cell assay.

In contrast to adenosine kinase, TbrGAPDH activity was not influenced by any of the compounds with respect to forward and reverse reaction (Fig. S5) when measured at concentrations up to 50 µM (limit given due to strong absorption at the wavelength applied), suggesting that TbrGAPDH could be considered as a false positive hit.

### Compound 1 is binding specifically to TbrAK

Specific binding of compound **1** with respect to the isolated proteins was analyzed by two methods. Thermal stability of proteins and protein complexes can be evaluated by CD spectroscopy. Due to the fact that the complex of a ligand bound to the native conformation of a protein will have a higher thermal stability than the empty protein [14], an increased melting point (T_m_) of the protein in presence of a ligand will give evidence for specific binding. Indeed, apo TbrAK melted at 43.1±0.4°C and was stabilized by the control compound adenosine with a ΔT_m_ of 7.6°C (T_m_ 50.7±0.2°C) (Table 1). In a similar way, compound **1** and **2** were able to increase thermal stability by a ΔT_m_ of 4.8°C (T_m_ 47.9±0.1°C) and a ΔT_m_ of 2.8°C (T_m_ of 45.9±0.4°C), respectively, thus confirming specific binding for compound **1** to the enzyme (Table 1). Compound **3**, **4**, and **5** did not stabilize the protein when added at 50 µM (Table 1).

**Table 1:** Thermal stability assay regarding TbrAK in absence and presence of compounds and substrates.

	T_m_ [°C]^a^	ΔT_m_ [°C]
TbrAK	43.1±0.4	–
TbrAK+compound **1**	47.9±0.1	4.8
TbrAK+compound **2**	45.9±0.4	2.8
TbrAK+compound **3**	42.8±0.2	−0.3
TbrAK+compound **4**	43.2±0.1	0.1
TbrAK+compound **5**	43.4±0.1	0.3
TbrAK+ATP	42.8±0.1	−0.3
TbrAK+adenosine	50.7±0.2	7.6

a Values represent the average of three independent experiments.

**Figure 4:**
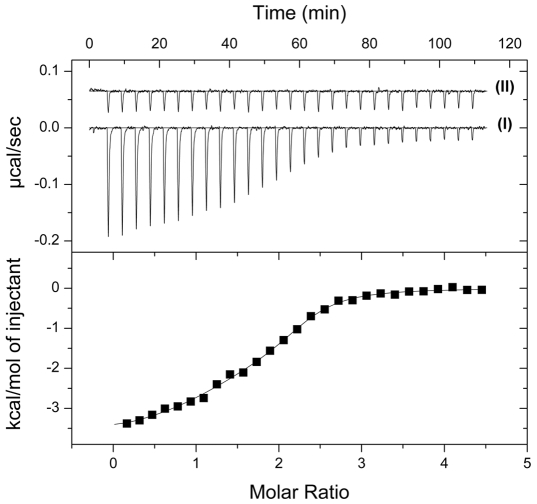
Specific binding of compound 1 to TbrAK measured by ITC. The top panel shows heat signals upon 27 injections of compound 1 (trace I) or control compound 5 (trace II) into the sample cell containing 7 µM TbrAK. The binding isotherm obtained by integration and normalization of the raw data and by correction for the heat of ligand dilution is shown on the lower panel. The solid line represents the non-linear least square fit based on a two-sites non-interacting binding model. Compound 1 binds to TbrAK via a high affinity binding site with a K_D_ of 75±20 nM and a ΔH_bind_ of −3.05±0.77 kcal/mol, and a low affinity site exhibiting a K_D_ of 497±34 nM and a ΔH_bind_ of −1.13±0.24 kcal/mol. No specific heat release was shown for the negative control (trace II). The mean of three independent experiments are reported.

As an orthogonal method to CD we used isothermal titration calorimetry (ITC) to analyze binding of compound **1** to TbrAK. The titration of TbrAK with compound **1** revealed a complex and enthalpy driven binding mode with two molecules of **1** binding to one molecule of enzyme. Exothermic binding heats were corrected for heats of dilution, integrated and plotted against the molar ratio of **1** and enzyme (trace I in Fig. 4). The binding isotherm was best described using a non-linear least square fit assuming a two-sites non-interacting binding model. Subsequent analysis revealed a high affinity binding site with a K_D_ of 75±20 nM and a ΔH_bind_ of −3.05±0.77 kcal/mol, while the low affinity site exhibited a K_D_ of 497±34 nM and a ΔH_bind_ of −1.13±0.24 kcal/mol. The negative control using compound **5** showed no specific heat release (see trace II in Fig. 4).

In contrast to TbrAK, TbrGAPDH was not significantly stabilized by any of the compounds, while in presence of the substrate NAD^+^ or DL-GAP thermal stability increased (see Table S1). In agreement with above TbrGAPDH activity measurement, no binding of the compounds to the protein was observed, clearly indicating that TbrGAPDH is a false positive hit. Therefore, this enzyme was not further validated.

### Compound 1 interferes with substrate-inhibition of TbrAK

**Figure 5:**
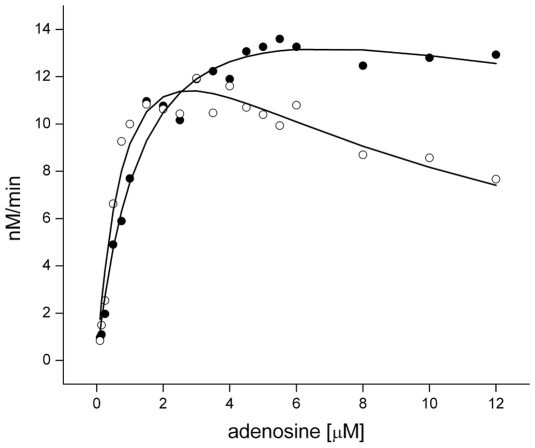
Representative plots for TbrAK kinetics (AMP formation) with respect to adenosine in absence (○) and presence (•) of compound 1 (33 µM). Adenosine strongly inhibits TbrAK at concentrations >2 µM and follows typical substrate-inhibition kinetics. In contrast, compound 1 abolishes substrate-inhibition. The solid lines indicate the fit of the raw data with the substrate-inhibition model (see Table 2 for fitting results).

A common property of adenosine kinases from various organisms is their control via a substrate-inhibition mechanism [15]–[18], and recently it was shown that *T. brucei* AK was inhibited by high adenosine concentrations to prevent non-physiologically high intracellular purine nucleotide levels [19]. This prompted us to analyze the influence of compound **1** toward TbrAK with respect to substrate transformation by determining the kinetic parameters in absence and presence of the activator. TbrAK activity was measured at increasing adenosine concentrations and a fixed concentration of 167 µM ATP. All data are given in Table 2. Adenosine kinetics in absence of compound **1** displayed non-hyperbolic progress plots (Fig. 5). After increasing activity up to a maximum at 2–3 µM adenosine, the enzyme activity declined at higher substrate concentrations which is a typical finding for substrate-inhibition. Thus the observed kinetic data were fit to the substrate-inhibition model [10]. Indeed, a good fit was obtained for all data points, yielding a K_m_ of 0.99±0.05 µM and a V_max_ of 19.60±0.33 nM/min. k_cat_ and catalytic efficiency were found to be 0.37±0.01 s^−1^ and 0.38±0.01 µM^−1^ s^−1^, respectively. TbrAK was inhibited by adenosine with a K_i_ of 6.1±1.4 µM. In contrast, in presence of compound **1** substrate-inhibition was strongly reduced (Fig. 5), and adenosine inhibited TbrAK with a more than ten-fold increased K_i_ value (78.4±2.2 µM). While K_m_ (0.65±0.04 µM), V_max_ (14.20±0.14 µM) and k_cat_ (0.0.27±0.01 s^−1^) were slightly reduced in presence of compound **1**, the catalytic efficency k_cat_/K_m_ (0.42±0.01 µM^−1^ s^−1^) did not change significantly (Table 2).

**Table 2:** Parameters derived from TbrAK kinetics with respect to adenosine in absence and presence of compound **1**.

	no compound	compound 1
K_m_ [µM]	0.99±0.05	0.65±0.04
V_max_ [nM/min]	19.60±0.33	14.20±0.14
k_cat_ [s^−1^]	0.37±0.01	0.27±0.01
k_cat_/K_m_ [µM^−1^ s^−1^]	0.38±0.01	0.42±0.01
K_i_ adenosine [µM]	6.1±1.4	78.4±2.2

The substrate-inhibition model was applied to fit the raw data; values represent the average of three independently recorded series with each data point measured in triplicate.

### Down-regulation of adenosine kinase in *T. b. brucei* counteracts compound 1 activity

**Figure 6:**
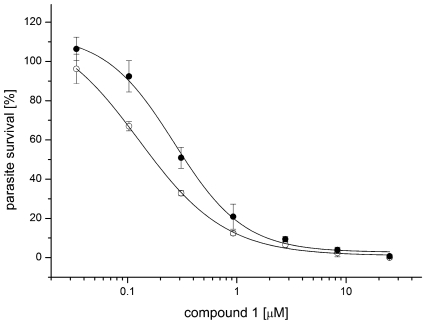
Dose-response curve for parasite growth in presence of compound 1. RNAi-mediated silencing of TbAK expression by tetracycline (•) reduces sensitivity of TbAK RNAi cells to compound 1 when compared to non-induced cells (○), raising the IC_50_ from 131±43 nM to 271±25 nM (two-tailed t-test, p<0.05).

The proposed mechanism of action for compound **1**, hyperactivation of TbrAK, implies that down-regulation of TbrAK activity counteracts compound **1** toxicity. This hypothesis was addressed by RNAi-mediated silencing of TbAK expression, using bloodstream-form *T. b. brucei* that express in a tetracycline-inducible manner a stem-loop construct targeted against TbAK [11]. TbAK knock-down has been confirmed recently on the RNA and protein level [11],[19]. The addition of tetracycline reduced the sensitivity of TbAK RNAi cells to compound **1**, raising the IC_50_ from 131±43 nM to 271±25 nM (two-tailed t-test, p<0.05; Fig. 6). The negative control (compound **5**) did not exhibit any IC_50_ difference in the induced and non-induced TbAK RNAi cells.

## Discussion

A chemical proteomics approach was applied to isolate and identify the cellular target of compound **1**. To this end, three of its amino-derivatives where immobilized, and the corresponding affinity matrixes were used to pull down potential targets. Indeed, this approach afforded multiple proteins. Most of them could be detected in both the control and the isolation experiment (Fig. 2) and thus could be considered as unspecific bound protein. Amongst other, human keratin was the predominant contamination which turned out to be present under any circumstances, and it was even found in new batches of matrix that were stored in sealed containers before first use. Remarkably, we observed differences regarding inter- and intrabatch reproducibility. Experiments were always reproducible with respect to background when the same batch of activated matrix was used while changing to a new batch led to altered background (see lane a in Fig. 2A,B,C). However, two distinct proteins not appearing in the control reaction were identified as TbrGAPDH and TbrAK, two trypanosomal enzymes that are involved in parasite glycolysis and purine salvage, respectively. Both enzymes were cloned and recombinantly expressed.

The subsequent chemical validation of both enzymes revealed that TbrGAPDH activity was neither affected by compound **1** (Fig. S5) nor specific binding of **1** toward the enzyme was observed (Table S1), suggesting TbrGAPDH to be a false positive hit. As a matter of fact, due the highly charged nature and its high abundance in the parasite this particular target has a strong tendency to be isolated by affinity chromatography [20]–[22].

In contrast and unexpectedly, the HPLC activity assay revealed that compound **1** strongly activated TbrAK activity. A similar effect was measured for compound **2**, while derivatives **3** and **4** only exhibited very low though significant activity toward TbrAK (Fig. S3). Compound **1**, **2** and **4** activated TbrAK in a concentration dependent manner, yielding an EC_50_ of 38.9±0.9 µM for compound **1** (Fig. 3). While the EC_50_ of **2** and **4** could not be determined due to solubility problems, it can be estimated that they would be higher than the one observed with compound **1** (Fig. 3). The observed data for compound **1** and **2** correlate well with formerly determined *in-vitro* data that showed an IC_50_ of 1.0 µM and 17.7 µM against blood stage *T.b. rhodesiense* [7], and also with the fact that compound **2** was able to bind the target. TbrAK activation by derivative **4** was concentration dependent (EC_50_>750 µM, Fig. 3) which corresponds very well to the low activation capacity at 50 µM (109±1%, Fig. S3), (see text below for discussion of this issue).

Specific binding could be verified for compounds **1** and **2** by the thermal stability assay as shown by the T_m_ increase (Table 1). For the least active compounds (**3** and **4**) no stabilization was observed. While this finding is in line with the lack of activity at all concentrations tested for compound **3**, it seems to contrast the concentration dependence of derivative **4**. However, taking into consideration the small activating effect, the concentration dependence and the unchanged T_m_ in presence of compound **4**, it is likely to assume that this derivative binds with low affinity to the target. Unfortunately, the limited solubility prevented further investigations at concentrations >750 µM regarding T_m_ as well as binding affinity due to the technical limits given by CD spectroscopy and ITC. As expected the negative control (compound **5**) did neither activate TbrAK nor improve thermal stability, giving further evidence for specific binding of compound **1** and **2**. In addition, binding of compound **1** to TbrAK could be confirmed by ITC which revealed enthalpy driven high affinity interaction. Interestingly, it appears that two molecules of the activator are bound per molecule of enzyme. Under the same conditions control compound **5** did not bind to the enzyme and produced only unspecific heat signals related to protein and ligand dilution in the sample cell (Fig. 4).

On the target level it is evident that the additional amino group in derivative **2** only moderately interferes with the activating effect, thus binding to TbrAK seems disturbed but still productive. In contrast, replacing the oxygen in the morpholine moiety by nitrogen (compound **4**) strongly increases EC_50_ at least 20 fold, suggesting that binding to TbrAK is severely impaired with this derivative. Finally, a substitution on the pyrazole moiety (compound **3**) leads to very low and concentration independent activity. These observations are well in line with the fact that none of these two derivatives stabilized TbrAK in the thermal stability assay (Table 1). On the parasite level the situation is different. Regarding derivative **3**, the very low activity toward the isolated enzyme while exhibiting an IC_50_ of 10.3 µM on the parasite [7], leads to the conclusion that toxicity is not conferred by TbrAK but by another yet to be determined target. For compound **4** the situation appears more complex. The observed concentration dependence gives strong evidence for it acting against TbrAK, but its potentially low affinity and the low activation capacity (EC_50_ >750 µM) toward the target would lead to reduced trypanocidal activity. However, derivative **4** is highly potent in the parasite assay (IC_50_ of 1.1 µM) [7]. It is therefore likely that the trypanocidal effect of compound **4** is based on a combination of target specific activity, and/or possible off-target activity, and increased general toxicity [7]. In addition, increased accumulation within the parasite compared to compound **1** could contribute to the strong effect, while reduced cell uptake may explain the difference in potency of derivative **2** on the isolated target. Thus, taking into consideration the above observations and the complete lack of activity of the control compound **5** in all experiments, we can state that the intact morpholine/pyrazole moiety represents an important part of the pharmacophore, while the introduction of basic amino groups may impair the activating effect due to altered physico-chemical properties on target and/or parasite level.

To further validate TbrAK as the potential target of compound **1** we investigated parasite sensitivity toward compound **1** under conditions of reduced intracellular adenosine kinase levels. To this end, parasite viability of a knock-down mutant was measured. Indeed, as expected for a mechanism of action based on overactivation of TbrAK by compound **1**, the sensitivity of TbrAK silenced parasite cells decreased as shown by the IC_50_ raising from 131±43 nM to 271±25 nM (two-tailed t-test, p<0.05). Although the applied tetracycline-inducible system is leaky and not capable to provide a complete knock-out [11] the observed difference is statistically significant, demonstrating that the toxic effect of compound **1** is adenosine kinase dependent. Although we cannot rule out that compound **1** could interact with other targets in the cell that may not be amenable to the pull down approach (e.g. cytoskeleton, DNA, interference with mitochondrial electron transport), the line of evidence strongly supports TbrAK to represent the putative cellular target.

A first step toward the elucidation of the mechanism of action with respect to the activating effect was accomplished by determination and analysis of kinetic parameters with respect to substrate transformation in absence and presence of the activator **1**. TbrAK activity is strongly inhibited by its substrate adenosine (K_i_ of 6.1±1.4 µM), thus it follows substrate-inhibition kinetics. This is a common characteristic of adenosine kinases isolated from various sources [15]–[18] and has also been described for TbrAK very recently [19]. Under the experimental conditions applied, the K_i_ of adenosine increased more than ten-fold to a value of 78.4±2.2 µM when compound **1** was present, resulting in strongly reduced substrate-inhibition (Fig. 5) while the kinetic parameters remained almost identical. Taken together, the results suggest that the mechanism for trypanocidal activity functions via hyperactivation of adenosine kinase. There are two principally different explanations for the toxic effect of hyperactivation. Uncontrolled TbrAK activity could lead to purine imbalance within the parasite, thus interfering with the vital purine salvage pathway, the nucleotide pool and subsequently nucleic acid formation. Precedence for this mechanism comes from *E. coli*, where high adenine concentrations cause a cytotoxic [ATP]/[GTP] imbalance [23]. Alternatively, excessive adenosine kinase activity may use up the existing adenosine/ATP pools and lead to adenosine depletion and ATP burn-out. Interestingly, cell death upon hydrolysis of ATP reserves due to mislocalization of glycosomal hexokinase to the cytosol has already been observed for *T. brucei* [24],[25].

Whereas hyperactivation as a mechanism of action is well known from drugs targeting cell signaling, e.g. acetylcholine receptor agonists, it represents a novel and hitherto unexplored concept for compounds targeting metabolic enzymes. The cytotoxic hyperactivation of adenosine kinase does not only provide an opportunity for the chemotherapy of sleeping sickness, but when explored against other pathogens or tumor cells, hyperactivation of metabolic key enzymes may well find further pharmacological applications.

## Supporting Information

Figure S1 Affinity matrix preparation. 4-[4-(5-morpholine-4-yl-1*H*-pyrazol-3-yl)-phenoxy]-phenylamine (2), 4-[5-morpholine-4-yl-3-(4-phenoxyphenyl)-pyrazol-1-yl]phenylamine (3) and 1-[5-(4-phenoxy-phenyl-2*H*-pyrazol-3-yl]piperazine (4) were coupled to epoxy-activated agarose consisting of a 12-atoms spacer (1,4-bis(2∶3-epoxypropoxy)butane) to form affinity matrices A, B, and C, respectively. The agarose beads are shown as spheres.
(0.04 MB PDF)

Figure S2 SDS-PAGE analysis of expression and purification of recombinant proteins. Panel A: TbrGAPDH, panel B: TbrAK. Lane 1: marker proteins; lane 2: soluble fraction of crude extract; lane 3: final pure protein. The expected molecular weights for TbrGAPDH (including His_6_-tag) and TbrAK are 41.2 kDa and 38.0 kDa, respectively. Molecular mass of the markers are shown on the left.
(0.14 MB PDF)

Figure S3 Activation properties of compounds 1 to 5 measured by HPLC and monitoring ADP formation and ATP consumption. TbrAK (0.7 µM) was incubated for 10 min at 37°C in absence or presence of 50 µM compound. Compound 1 and 2 lead to strong TbrAK activation with compound 1 showing up to a 2.5 fold increase. The corresponding values are: 100±2% without compound (TbrAK), 245±3% (compound 1), 174±5% (compound 2), 110±1% (compound 3), 109±1% (compound 4), and 102±2% for the negative control (compound 5). Values are reported as % activity derived from ADP/ATP ratios. For comparative reasons the activity recorded in absence of compound (column labeled TbrAK) was set to 100%. The mean of three independent experiments is reported.
(0.02 MB PDF)

Figure S4 Concentration dependence of the activation effect of compound 1 analyzed by a radiometric assay. Increasing concentrations of compound 1 yield a sigmoid saturation curve for TbrAK activation with an EC_50_ value of 38±12 nM. Values are reported as % activity derived from the transformation rate. For comparative reasons the activity recorded in absence of compound was set to 100%. The mean of four independent measurements is reported.
(0.02 MB PDF)

Figure S5 UV-spectroscopic analysis of TbrGAPDH activity (reverse reaction) in absence and presence of compounds 1 to 5 at 50 µM concentration. TbrGAPDH (60 nM) was incubated for 10 min at 25°C in absence or presence of each compound. None of the compounds either showed significantly increased or decreased activity at concentrations up to 50 µM. Values are reported as % activity derived from the transformation rate. For comparative reasons the activity recorded in absence of compound (column labeled TbrGAPDH) was set to 100%. The mean of three independent experiments is reported.
(0.02 MB PDF)

Table S1 Thermal stability assay regarding TbrGAPDH in absence and presence of compounds and substrates.
(0.07 MB PDF)

Text S1 Supporting information with respect to origin of supplies, protocols for purification of recombinant proteins, the spectroscopic assay for TbrGAPDH, the radiometric assay for TbrAK, and the thermal denaturation assay.
(0.09 MB PDF)
