# Supplementary material for: Adenosine Kinase of T. b. rhodesiense Identified as the Putative Target of 4-[5-(4-phenoxyphenyl)-2H-pyrazol-3-yl]morpholine Using Chemical Proteomics
Source: PLoS Negl Trop Dis. 2009 Aug 25;3(8):e506. doi: 10.1371/journal.pntd.0000506 (PMC2724708; doi:10.1371/journal.pntd.0000506)
Supplement: Figure S1 — Affinity matrix preparation. 4-[4-(5-morpholine-4-yl-1H-pyrazol-3-yl)-phenoxy]-phenylamine (2), 4-[5-morpholine-4-yl-3-(4-phenoxyphenyl)-pyrazol-1-yl]phenylamine (3) and 1-[5-(4-phenoxy-phenyl-2H-pyrazol-3-yl]piperazine (4) were coupled to epoxy-activated agarose consisting of a 12-atoms spacer (1,4-bis(2∶3-epoxypropoxy)butane) to form affinity matrices A, B, and C, respectively. The agarose beads are shown as spheres. (0.04 MB PDF) [file pntd.0000506.s001.pdf]

Supporting Information Figure S1

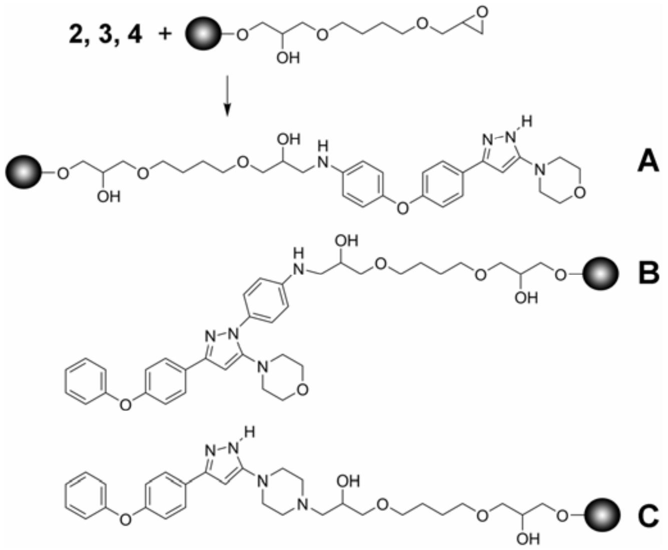

Figure S1. Affinity matrix preparation. 4-[4-(5-morpholine-4-yl-1*H*-pyrazol-3-yl)-phenoxy]-phenylamine (**2**), 4-[5-morpholine-4-yl-3-(4-phenoxyphenyl)-pyrazol-1-yl]phenylamine (**3**) and 1-[5-(4-phenoxy-phenyl-2*H*-pyrazol-3-yl]piperazine (**4**) were coupled to epoxy-activated agarose consisting of a 12-atoms spacer (1,4-bis(2:3-epoxypropoxy)butane) to form affinity matrices **A**, **B**, and **C**, respectively. The agarose beads are shown as spheres.
