# Supplementary material for: Adenosine Kinase of T. b. rhodesiense Identified as the Putative Target of 4-[5-(4-phenoxyphenyl)-2H-pyrazol-3-yl]morpholine Using Chemical Proteomics
Source: PLoS Negl Trop Dis. 2009 Aug 25;3(8):e506. doi: 10.1371/journal.pntd.0000506 (PMC2724708; doi:10.1371/journal.pntd.0000506)
Supplement: Figure S2 — SDS-PAGE analysis of expression and purification of recombinant proteins. Panel A: TbrGAPDH, panel B: TbrAK. Lane 1: marker proteins; lane 2: soluble fraction of crude extract; lane 3: final pure protein. The expected molecular weights for TbrGAPDH (including His6-tag) and TbrAK are 41.2 kDa and 38.0 kDa, respectively. Molecular mass of the markers are shown on the left. (0.14 MB PDF) [file pntd.0000506.s002.pdf]

Supporting Information Figure S2

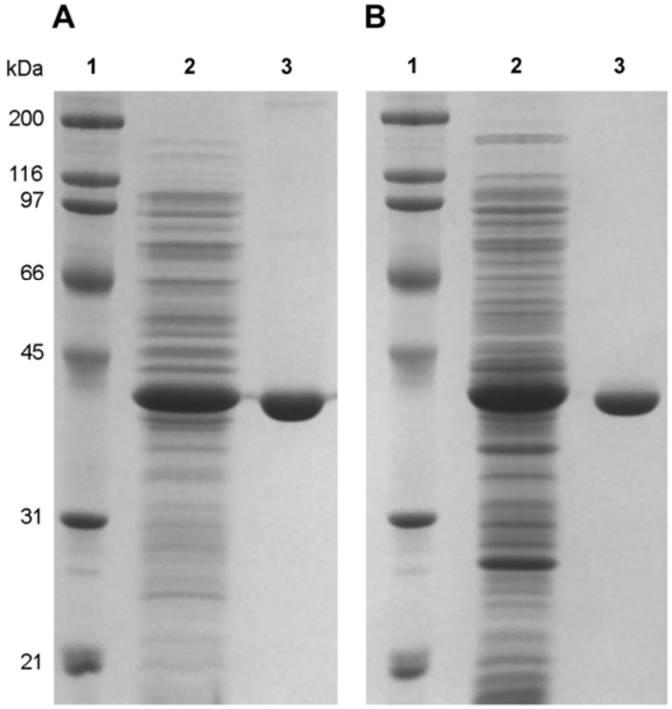

Figure S2. SDS-PAGE analysis of expression and purification of recombinant proteins. Panel A: TbrGAPDH, panel B: TbrAK. Lane 1: marker proteins; lane 2: soluble fraction of crude extract; lane 3: final pure protein. The expected molecular weights for TbrGAPDH (including His_6-tag) and TbrAK are 41.2 kDa and 38.0 kDa, respectively. Molecular mass of the markers are shown on the left.
