# Supplementary material for: Adenosine Kinase of T. b. rhodesiense Identified as the Putative Target of 4-[5-(4-phenoxyphenyl)-2H-pyrazol-3-yl]morpholine Using Chemical Proteomics
Source: PLoS Negl Trop Dis. 2009 Aug 25;3(8):e506. doi: 10.1371/journal.pntd.0000506 (PMC2724708; doi:10.1371/journal.pntd.0000506)
Supplement: Figure S3 — Activation properties of compounds 1 to 5 measured by HPLC and monitoring ADP formation and ATP consumption. TbrAK (0.7 µM) was incubated for 10 min at 37°C in absence or presence of 50 µM compound. Compound 1 and 2 lead to strong TbrAK activation with compound 1 showing up to a 2.5 fold increase. The corresponding values are: 100±2% without compound (TbrAK), 245±3% (compound 1), 174±5% (compound 2), 110±1% (compound 3), 109±1% (compound 4), and 102±2% for the negative control (compound 5). Values are reported as % activity derived from ADP/ATP ratios. For comparative reasons the activity recorded in absence of compound (column labeled TbrAK) was set to 100%. The mean of three independent experiments is reported. (0.02 MB PDF) [file pntd.0000506.s003.pdf]

Supporting Information Figure S3

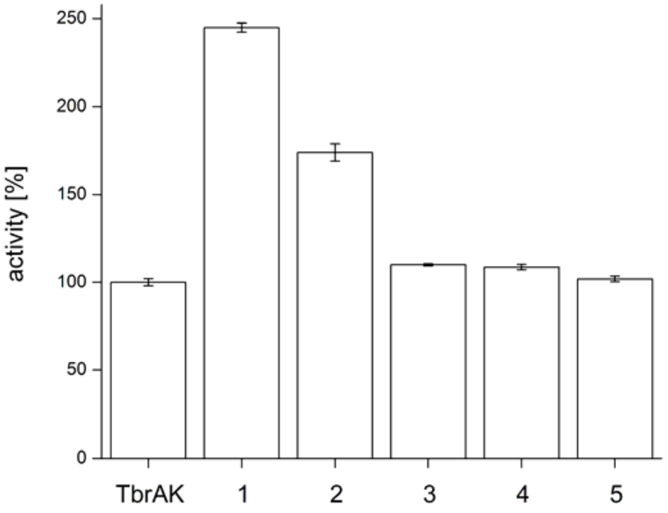

Figure S3. Activation properties of compounds 1 to 5 measured by HPLC and monitoring ADP formation and ATP consumption. TbrAK (0.7 µM) was incubated for 10 min at 37°C in absence or presence of 50 µM compound. Compound **1** and **2** lead to strong TbrAK activation with compound **1** showing up to a 2.5 fold increase. The corresponding values are: 100 ± 2 % without compound (TbrAK), 245 ± 3 % (compound **1**), 174 ± 5% (compound **2**), 110 ± 1 % (compound **3**), 109 ± 1 % (compound **4**), and 102 ± 2 % for the negative control (compound **5**). Values are reported as % activity derived from ADP/ATP ratios. For comparative reasons the activity recorded in absence of compound (column labeled TbrAK) was set to 100 %. The mean of three independent experiments is reported.
