# Supplementary material for: Adenosine Kinase of T. b. rhodesiense Identified as the Putative Target of 4-[5-(4-phenoxyphenyl)-2H-pyrazol-3-yl]morpholine Using Chemical Proteomics
Source: PLoS Negl Trop Dis. 2009 Aug 25;3(8):e506. doi: 10.1371/journal.pntd.0000506 (PMC2724708; doi:10.1371/journal.pntd.0000506)
Supplement: Figure S4 — Concentration dependence of the activation effect of compound 1 analyzed by a radiometric assay. Increasing concentrations of compound 1 yield a sigmoid saturation curve for TbrAK activation with an EC50 value of 38±12 nM. Values are reported as % activity derived from the transformation rate. For comparative reasons the activity recorded in absence of compound was set to 100%. The mean of four independent measurements is reported. (0.02 MB PDF) [file pntd.0000506.s004.pdf]

Supporting Information Figure S4

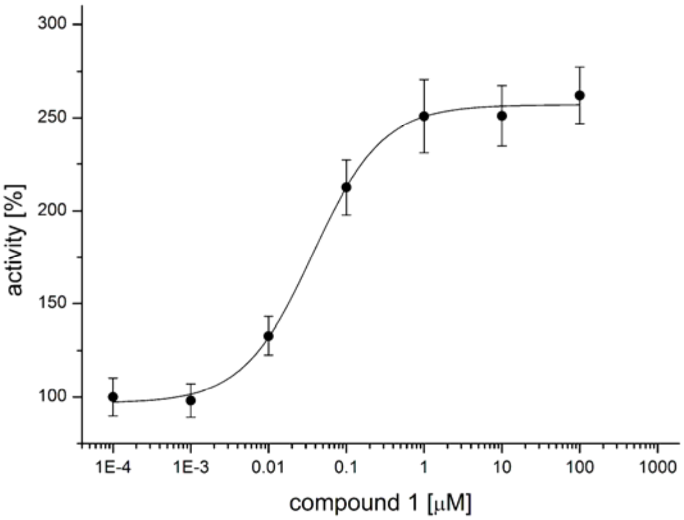

Figure S4. Concentration dependence of the activation effect of compound 1 analyzed by a radiometric assay. Increasing concentrations of compound **1** yield a sigmoid saturation curve for TbrAK activation with an EC_{50} value of 38 ± 12 nM. Values are reported as % activity derived from the transformation rate. For comparative reasons the activity recorded in absence of compound was set to 100 %. The mean of four independent measurements is reported.
