# Supplementary material for: Adenosine Kinase of T. b. rhodesiense Identified as the Putative Target of 4-[5-(4-phenoxyphenyl)-2H-pyrazol-3-yl]morpholine Using Chemical Proteomics
Source: PLoS Negl Trop Dis. 2009 Aug 25;3(8):e506. doi: 10.1371/journal.pntd.0000506 (PMC2724708; doi:10.1371/journal.pntd.0000506)
Supplement: Figure S5 — UV-spectroscopic analysis of TbrGAPDH activity (reverse reaction) in absence and presence of compounds 1 to 5 at 50 µM concentration. TbrGAPDH (60 nM) was incubated for 10 min at 25°C in absence or presence of each compound. None of the compounds either showed significantly increased or decreased activity at concentrations up to 50 µM. Values are reported as % activity derived from the transformation rate. For comparative reasons the activity recorded in absence of compound (column labeled TbrGAPDH) was set to 100%. The mean of three independent experiments is reported. (0.02 MB PDF) [file pntd.0000506.s005.pdf]

Supporting Information Figure S5

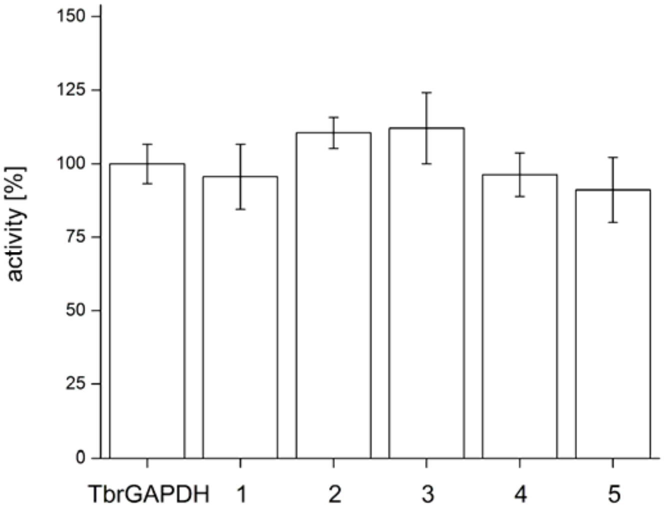

Figure S5. UV-spectroscopic analysis of TbrGAPDH activity (reverse reaction) in absence and presence of compounds 1 to 5 at 50 µM concentration. TbrGAPDH (60 nM) was incubated for 10 min at 25°C in absence or presence of each compound. None of the compounds either showed significantly increased or decreased activity at concentrations up to 50 µM. Values are reported as % activity derived from the transformation rate. For comparative reasons the activity recorded in absence of compound (column labeled TbrGAPDH) was set to 100 %. The mean of three independent experiments is reported.
