# Supplementary material for: Adenosine Kinase of T. b. rhodesiense Identified as the Putative Target of 4-[5-(4-phenoxyphenyl)-2H-pyrazol-3-yl]morpholine Using Chemical Proteomics
Source: PLoS Negl Trop Dis. 2009 Aug 25;3(8):e506. doi: 10.1371/journal.pntd.0000506 (PMC2724708; doi:10.1371/journal.pntd.0000506)
Supplement: Table S1 — Thermal stability assay regarding TbrGAPDH in absence and presence of compounds and substrates. (0.07 MB PDF) [file pntd.0000506.s006.pdf]

Supporting Information Table S1

Table S1. Thermal stability assay regarding TbrGAPDH in absence and presence of compounds and substrates.

	T_m [°C] [a]	ΔT_m [°C]
TbrGAPDH	47.6 ± 0.3	–
TbrGAPDH + compound **1**	46.8 ± 0.1	- 0.8
TbrGAPDH + compound **2**	47.0 ± 0.4	- 0.6
TbrGAPDH + compound **3**	45.5 ± 0.6	- 2.1
TbrGAPDH + compound **4**	47.0 ± 0.1	- 0.6
TbrGAPDH + compound **5**	46.7 ± 0.2	- 0.9
TbrGAPDH + NAD^+	55.6 ± 0.2	8.0
TbrGAPDH + DL-GAP	50.0 ± 0.7	2.4

[a]Values represent the average of at least two experiments.
