# Supplementary material for: Adenosine Kinase of T. b. rhodesiense Identified as the Putative Target of 4-[5-(4-phenoxyphenyl)-2H-pyrazol-3-yl]morpholine Using Chemical Proteomics
Source: PLoS Negl Trop Dis. 2009 Aug 25;3(8):e506. doi: 10.1371/journal.pntd.0000506 (PMC2724708; doi:10.1371/journal.pntd.0000506)
Supplement: Text S1 — Supporting information with respect to origin of supplies, protocols for purification of recombinant proteins, the spectroscopic assay for TbrGAPDH, the radiometric assay for TbrAK, and the thermal denaturation assay. (0.09 MB PDF) [file pntd.0000506.s007.pdf]

Supporting Information Text S1

Materials and instrumentation

Epoxy-activated agarose (E6632) as well as protease inhibitor cocktail (P8340) were purchased from Sigma. Silver-staining kit was purchased from Invitrogen. gDNA was kindly provided from the Swiss Tropical Institute from Switzerland. All cloning steps were performed in *E. coli* Nova Blue or DH5α cells (Stratagene). Expression was conducted in *E. coli* BL21(DE3)-CodonPlus-RIL or BL21(DE3) cells (Stratagene). Sources of supplies are as follows: pET28a and pET30b vectors from Novagen (the latter modified by changing the NcoI position); Taq polymerase from Qbiogene; dNTP from Promega; T4 DNA ligase and restriction enzymes from New England Biolabs; plasmid extraction kits from Sigma; Terrific broth (TB) medium from Difco; HisTrap HP, HiTrap Q XL anion exchange and Superdex 75 gel filtration columns from Amersham Biosciences; PD-10 desalting columns from GE Healthcare. An Aekta FPLC (Amersham Biosciences) was used for protein purification. Spectrophotometric measurements were carried out on a Cary 50 Varian Spectrophotometer. HPLC separations were performed using a RP-18e (5 μm) cartridge (LiChroCART® 250-4) protected with a guard column on a Merck instrument. CD and ITC measurements were accomplished on Jasco J-815 CD Spectrophotometer and Omega titration microcalorimeter (VP-ITC Micro-Calorimeter from MicoCal™), respectively. [2-^{3}H]Adenosine [15 Ci/mmol (1 mCi = 37 MBq)] was purchased from Amersham GE Healthcare. DE81 paper to perform the anion exchange was used from Whatman. Ultima Gold scintillation liquid was from Perkin Elmer and the counting of the scintillation was performed on a LKB Wallac 1214 Rackbeta beta counter. Apart from TCEP (Molecular Probes), all other supplies were reagent grade or better and purchased from Sigma.

Cloning, expression and purification of TbrAK

The peptides resulting from tryptic digestion and mass spectrometry analysis corresponded to *T. b. rhodesiense* adenosine kinase (TbrAK; Swiss-Prot/TrEMBL entry code Q584S0). The coding region of TbrAK was amplified from *T. b. rhodesiense* gDNA by PCR using forward primer 5`-TTTTT<u>CCATGG</u>CATCCGCTCCTCTGAGG-GTATACGTTCA-3' and reverse primer 5`-AA<u>GAATTC</u>CACCATCGAAATCATATCAACCACCGTATTTGG-3'. NcoI and EcoRI restriction sites implemented for cloning are underlined. The reaction mixture (50 μl)

contained 100 ng of gDNA, 50 mM Tris/HCl (pH 9.1 at 25°C), 0.15 % BSA, 16 mM $(NH_4)_2SO_4$, 3.5 mM $MgCl_2$, 2.5 % DMSO, each dNTP at 500 μM, 50 pmol of each oligonucleotide primer and 5 units of Taq polymerase. The PCR program consisted of an initial 2 min denaturation step (94°C), followed by 25 cycles of 94°C for 30 s, 52°C for 30 s, 60°C for 3 min, and a final polishing step at 60°C for 5 min. The amplified gene was digested with NcoI and EcoRI and ligated into a modified pET30b overexpression vector using T4 DNA ligase. The plasmid was transformed into $CaCl_2$ competent DH5α. Positive clones were identified by restriction digestion and automated sequencing to verify authenticity.

The pET30b-TbrAK expression clone was designed to express TbrAK as an N-terminal His_6-fusion protein with an expected molecular mass of 39.7 kDa. The final plasmid was transformed into BL21(DE3)-CodonPlus-RIL expression strain, and the cells were grown in TB medium supplemented with kanamycin (100 μg/ml) and chloramphenicol (34 μg/ml) overnight at 37°C. Then the temperature was lowered to 12°C and the protein expression was induced with 1 mM IPTG for 72 h. Cells were harvested, resuspended in lysis buffer (20 mM Tris, 500 mM NaCl, 20 mM imidazole, pH 8) supplemented with DNase, and disrupted using a French press. The filtered supernatant was applied on a 5 ml HisTrap HP chelating column, washed with lysis buffer, and the protein was eluted with a linear imidazole gradient from 96 % buffer A (20 mM Tris, 500 mM NaCl, pH 8) to 100 % buffer B (20 mM Tris, 500 mM NaCl, 500 mM imidazole, pH 8) in 20 column volumes at a flow rate of 2 ml/min. Fractions containing TbrAK were pooled, 20 % glycerol and 2 mM $CaCl_2$ added, and the His_6-tag was removed using thrombin over night at 16°C. The digested protein was further diluted to give a NaCl concentration of 50 mM to load it on a 5 ml HiTrap Q XL ionic exchange column. The protein was eluted with a linear NaCl gradient using buffer C (20 mM Tris, pH 7.5) and buffer D (20 mM Tris, 1 M NaCl, pH 7.5) over 4 column volumes. The fractions containing the TbrAK were pooled, concentrated and applied on a Superdex 75 column equilibrated with buffer E1 (20 mM Hepes, 150 mM NaCl, pH 7.0) or buffer E2 (20 mM Tris, 150 mM NaCl, pH 7.5, 5 % glycerol). Total protein concentration was determined by the method of Bradford using bovine serum albumin as a standard [1].

Cloning, expression and purification of TbrGAPDH

The plasmid containing the *gapdh* gen (Swiss-Prot/TrEMBL entry code G3PG_TRYBB) and TbrGAPDH antibodies were kindly provided by Prof. Paul A. M. Michels (University of Louvain, Brussels, Belgium). The *gapdh* gen was cut out from the original plasmid using NdeI and EcoRI and subcloned into pET28a. Authenticity was verified by restriction digestion and automated sequencing. The expression clone was designed to express TbrGAPDH as an N-terminal His_6-fusion protein with an expected molecular mass of 41.2 kDa. The final plasmid was introduced into BL21(DE3) expression strain, and cells grown in LB medium supplemented with kanamycin (100 µg/ml) overnight at 37°C. After having lowered the temperature to 30°C, protein expression was induced with 0.4 mM IPTG for 5 to 7 h. The protein was purified as outlined for TbrAK, with the modification that the column was washed with lysis buffer, then buffer A (20 mM Tris, 500 mM NaCl, 250 mM imidazole, pH 8.0), and finally was eluted directly using buffer B (20 mM Tris, 500 mM NaCl, 500 mM imidazole, pH 8.0). Due pronounced instability the His_6-tag was not removed, but the protein was immediately exchanged into buffer C (20 mM Hepes, 150 mM NaCl, pH 7.0) using a PD-10 column. All measurements were done using freshly prepared and directly used enzyme. Total protein concentration was determined by the method of Bradford using bovine serum albumin as a standard [1].

Spectroscopic activity assay for TbrGAPDH

TbrGAPDH catalyzes the reversible conversion of glyceraldehyde-3-phosphate to 1,3-bisphospho-glycerate in the presence of NAD^+ and inorganic phosphate, thereby forming NADH, spectrophotometrically measured at 340nm over 10 min. Activity assays for both the forward and the reverse reaction were used. The forward reaction assay was performed at 25°C in a volume of 0.4 ml, containing 50 mM Bicine (pH 9), 150 mM NaCl, 1 mM EDTA, 1 mM NAD^+, 10 mM NaH_2PO_4, and 1 mM DL-glyceraldehyde-3-phosphate (DL-GAP). Compounds tested were dissolved in DMSO. The concentration of DMSO in the reaction cuvette was kept at 1 %. The reaction was started by addition of 1 µg enzyme (final concentration of 60 nM). Control reactions were run in the absence of compounds **1-5** but with 1 % DMSO. To determine activity of the reverse reaction 1,3-bisphosphoglycerate was generated in situ from glycerate-3-phosphate and ATP by 3-phosphoglycerate kinase and NADH oxidation was monitored at 340 nm. The reaction mixture contained 50 mM Mes (pH 7), 150 mM NaCl, 0.1 mM NADH, 10 mM

$MgSO_4$, 1 mM ATP, 1 mM glycerate-3-phosphate, and 0.06 units 3-phopspolycerate kinase according to supplier's instructions (Sigma). The reaction was started by addition of 1 µg TbrGAPDH at 25°C in a total volume of 0.4 ml.

Thermal denaturation

Thermal unfolding of TbrGAPDH and TbrAK was studied by circular dichroism (CD) spectroscopy at 220 nm within the temperature range of 20–90°C using a heating rate of 40°C/h. TbrAK in buffer E1 (20 mM Hepes, 150 mM NaCl, pH 7.0) was used at 0.5 mg/ml (final concentration 13 µM). All measurements were done in a 0.5 mm cell and contained 0.1 % DMSO and 2.5 mM EDTA to prevent enzyme activity. Thermal stability was analyzed in presence of 500 µM substrate to record the maximum stability limit, whereas compounds **1** to **5** were used at a concentration of 50 µM, being the highest concentration achievable for compound **2**, to allow comparison of all compounds at the same concentration. For evaluation of the CD spectra, the buffer spectrum was subtracted from the protein CD spectrum. All thermal unfolding curves were fitted to a two-state model that had been published recently [2]. The mean of three independent experiments are reported.

Radiometric assay for EC_{50} determination

The EC_{50} for hyperactivation of TbrAK by compound 1 was obtained by monitoring the conversion of [2-^{3}H]adenosine to [2-^{3}H]AMP. Reactions were executed at 37°C in a final volume of 30 µl containing 20 mM Hepes, 50 mM NaCl, 167 µM ATP, 167 µM $MgCl_2$, 0.33 % BSA, and 1.0 % DMSO. The concentration of [2-^{3}H]adenosine was kept constant at 12 µM while compound **1** was varied in the range of 0-100 µM. The reaction was started by the addition of 1 ng (0.88 nM) TbrAK. The reactions were incubated and 5 µl aliquots were spotted on 5 mm diameter DE81-cellulose disks placed in a 96-well plate to stop the reaction. The disks were washed 3 times with 250 µl 5 mM ammonium formate, once with H_2O, transferred to scintillation vials, and then soaked with 2 ml of the displacement solution (100 mM HCl, 200 mM KCl) and gently shaken for 1 minute to elute the phosphorylated product [3]. After adding 10 ml scintillation liquid, the samples were counted. For comparative reasons the activity recorded in absence of compound was set to 100 %. The mean of four independent measurements is reported.

References

1. Bradford MM (1976) A rapid and sensitive method for the quantitation of microgram quantities of protein utilizing the principle of protein-dye binding. Anal Biochem 72: 248-254.
2. Birringer MS, Perozzo R, Kut E, Stillhart C, Surber W, et al. (2006) High-level expression and purification of human thymidine kinase 1: quaternary structure, stability, and kinetics. Protein Expr Purif 47: 506-515.
3. Ren H, Wang L, Bennett M, Liang Y, Zheng X, et al. (2005) The crystal structure of human adenylate kinase 6: an adenylate kinase localized to the cell nucleus. Proc Natl Acad Sci USA 102: 303-308.
